# Effects on mortality of a nutritional intervention for malnourished HIV-infected adults referred for antiretroviral therapy: a randomised controlled trial

**DOI:** 10.1186/s12916-014-0253-8

**Published:** 2015-01-28

**Authors:** NUSTART (Nutritional Support for Africans Starting Antiretroviral Therapy) Study Team, Suzanne Filteau, George PrayGod, Lackson Kasonka, Susannah Woodd, Andrea M Rehman, Molly Chisenga, Joshua Siame, John R Koethe, John Changalucha, Denna Michael, Jeremiah Kidola, Daniela Manno, Natasha Larke, Daniel Yilma, Douglas C Heimburger, Henrik Friis, Paul Kelly

**Affiliations:** Faculty of Epidemiology and Population Health, London School of Hygiene and Tropical Medicine, Keppel Street, London, WC1E 7HT UK; Mwanza Research Centre, National Institute for Medical Research, Isamilo road, P.O.Box1462, Mwanza, Tanzania; University Teaching Hospital, Nationalist Road, Lusaka, Zambia; Vanderbilt Institute of Global Health, Vanderbilt University School of Medicine, 2525 West End Avenue, Nashville, TN USA; Jimma University Specialised Hospital, PO Box 574, Jimma, Ethiopia; University of Copenhagen, Copenhagen, Rolighedsvej 30, DK-1958 Frederiksberg, Denmark; Barts & The London School of Medicine, Queen Mary University of London, 4 Newark Street, London, UK

**Keywords:** Antiretroviral therapy, Body mass index, Electrolytes, HIV, Malnutrition, Micronutrients

## Abstract

**Background:**

Malnourished HIV-infected African adults are at high risk of early mortality after starting antiretroviral therapy (ART). We hypothesized that short-course, high-dose vitamin and mineral supplementation in lipid nutritional supplements would decrease mortality.

**Methods:**

The study was an individually-randomised phase III trial conducted in ART clinics in Mwanza, Tanzania, and Lusaka, Zambia. Participants were 1,815 ART-naïve non-pregnant adults with body mass index (BMI) <18.5 kg/m^2^ who were referred for ART based on CD4 count <350 cells/μL or WHO stage 3 or 4 disease. The intervention was a lipid-based nutritional supplement either without (LNS) or with additional vitamins and minerals (LNS-VM), beginning prior to ART initiation; supplement amounts were 30 g/day (150 kcal) from recruitment until 2 weeks after starting ART and 250 g/day (1,400 kcal) from weeks 2 to 6 after starting ART. The primary outcome was mortality between recruitment and 12 weeks of ART. Secondary outcomes were serious adverse events (SAEs) and abnormal electrolytes throughout, and BMI and CD4 count at 12 weeks ART.

**Results:**

Follow-up for the primary outcome was 91%. Median adherence was 66%. There were 181 deaths in the LNS group (83.7/100 person-years) and 184 (82.6/100 person-years) in the LNS-VM group (rate ratio (RR), 0.99; 95% CI, 0.80–1.21; *P* = 0.89). The intervention did not affect SAEs or BMI, but decreased the incidence of low serum phosphate (RR, 0.73; 95% CI, 0.55–0.97; *P* = 0.03) and increased the incidence of high serum potassium (RR, 1.60; 95% CI, 1.19–2.15; *P* = 0.002) and phosphate (RR, 1.23; 95% CI, 1.10–1.37; *P* <0.001). Mean CD4 count at 12 weeks post-ART was 25 cells/μL (95% CI, 4–46) higher in the LNS-VM compared to the LNS arm (*P* = 0.02).

**Conclusions:**

High-dose vitamin and mineral supplementation in LNS, compared to LNS alone, did not decrease mortality or clinical SAEs in malnourished African adults initiating ART, but improved CD4 count. The higher frequency of elevated serum potassium and phosphate levels suggests high-level electrolyte supplementation for all patients is inadvisable but the addition of micronutrient supplements to ART may provide clinical benefits in these patients.

**Trial registration:**

PACTR201106000300631, registered on 1^st^ June 2011.

## Background

The last decade has seen great advances in expanding access to antiretroviral therapy (ART) for HIV-infected Africans. However, high mortality in the first few months of ART remains a major concern [1,2]. Consistent risk factors for early mortality include low CD4 count, advanced WHO disease stage, the presence of opportunistic infections such as tuberculosis (TB), and malnutrition, usually indicated by low body mass index (BMI) [1,3,4].

Food insecurity, though widespread in much of Africa, is unlikely to be the main cause of low BMI among HIV-infected Africans. Asymptomatic HIV infection increases basal energy requirements by approximately 10%, and up to 30% among those with opportunistic infections, while intestinal malabsorption, also frequently observed in HIV, reduces nutrient uptake from food [5]. Anorexia, common among HIV patients, is probably the most important contributor to loss of cell mass and prevents weight regain until appetite returns [6]. A belief that managing infections through ART and other anti-microbial treatment will be sufficient to reverse the nutritional deficits may have contributed to the neglect of nutrition in HIV treatment policies. This view ignores a large literature on management of malnutrition among a different group: young children [7].

Even if malnutrition is secondary to infection, it requires careful nutritional intervention. Although intensive feeding of malnourished children to permit regain of weight might seem the obvious intervention, there is evidence that this is associated with increased mortality [7]. This is because severe malnutrition is associated with metabolic abnormalities, the hallmark of which is hypophosphataemia, which is often accompanied by disordered sodium/potassium balance and oedema. Recent evidence suggests that low plasma phosphate is also present among malnourished Africans starting ART and is an independent risk factor for early mortality [8,9].

Malnutrition is also associated with altered iron metabolism and provision of iron before metabolic iron-control mechanisms are restored may result in increased infections or oxidative stress [10]. Current practice is to treat severely malnourished children in two stages: first a stabilisation phase during which infections are cleared and metabolic abnormalities are reversed, and only secondly, a recovery phase during which weight gain is promoted [7]. Treatment of severe malnutrition in adults, other than those with prolonged illness in high-income countries, has been studied less often than treatment of malnourished children. During the 1992 famine in Somalia, Collins et al. [11] found that, similar to treatment of children, starting adults with a low protein and lower calorie diet intervention was associated with lower mortality than when using a high protein diet. A non-randomised addition of higher amounts of minerals to the low protein diet had no additional benefit.

There has been little controlled research on nutritional interventions for malnourished, HIV-infected Africans [12]. A recent Cochrane review [13] found few placebo-controlled trials, small sample sizes, and few benefits of macronutrient interventions for HIV. A trial excluded from the review because it compared two dietary supplements, rather than having a placebo control, found increased BMI in Malawian adults after 14 weeks of supplementation with nutrient-dense food [14], but not 9 months after stopping supplementation [15] and no effect on the high mortality of the patients [14]. A study conducted since the review found that lipid-based nutritional supplements (LNS) given at the start of ART, compared with LNS given 3 months later, increased early gains in BMI, lean mass, and grip strength [16]. In earlier work, we found preliminary evidence that micronutrient supplementation reduced mortality [17]; sample size was small, a feature common to all multiple micronutrient trials for HIV-infected adults in a systematic review [18]. Nevertheless, this review documents evidence that provision of additional micronutrients may have some benefits for HIV patients. The Nutritional Support for Adults Starting Antiretroviral Therapy (NUSTART) trial was designed to build on these trials and to incorporate two key lessons learned from treating childhood malnutrition: stabilisation of nutritional metabolism before introducing high energy supplementation, and avoidance of iron in the early stages. We hypothesised that provision of vitamins and minerals and use of a two-stage intervention with stabilisation and recovery stages would decrease the early mortality of malnourished adults starting ART.

## Methods

### Design

The NUSTART study was a blinded phase III individually-randomised controlled trial comparing in a two-stage protocol vitamin and mineral supplements in a lipid-based nutritional supplement (LNS-VM) with control LNS administered from recruitment at referral for ART until 6 weeks after starting ART. The primary outcome was mortality between recruitment and 12 weeks post-ART initiation. Secondary outcomes presented here are other serious adverse events (SAEs) and BMI, and CD4 count at 12 weeks. The trial was registered on the Pan-African Clinical Trials Register as PACTR201106000300631 (May 31, 2011).

### Setting

The study was conducted from August 2011 to December 2013 at two sites: the National Institute for Medical Research, Mwanza, Tanzania, and the University Teaching Hospital, Lusaka, Zambia. In Mwanza, patients were screened at six peripheral clinics and recruitment was conducted at a research clinic located at the Sekou Toure Regional Hospital. In Lusaka, patients were recruited from six peripheral clinics which referred to the University Teaching Hospital. At both sites, this resulted in patients with a wide range of socioeconomic and nutritional backgrounds. Previous work by our group at both sites confirmed that micronutrient deficiencies were prevalent and that multiple micronutrient supplementation could provide benefits [19-21]. HIV prevalence among adults in the Mwanza region is about 6% [22] and in Lusaka about 20% [23]. In both countries, at the time of the trial, ART was provided free for those with either CD4 lymphocyte count <350 cells/μL or WHO Stage 3 or 4 disease. About a third of patients starting ART in both countries have a BMI <18.5 kg/m^2^ [3,4].

### Participants

Inclusion criteria were at least 18 years old, ART-naive (except for standard regimens to prevent maternal-to-child HIV transmission), BMI <18.5 kg/m^2^, requiring ART as determined by CD4 count <350 cells/μL or stage 3 or 4 disease, willing to undertake intensive ART follow-up in the study clinic, and providing written (or thumbprint if unable to write) informed consent. In the presence of oedema, patients with BMI <20 kg/m^2^ were considered; BMI was re-measured after loss of oedema, and the patient considered eligible if BMI was <18.5 kg/m^2^ and ART had not yet been initiated. Exclusion criteria were participation in a potentially conflicting research protocol or self-reported pregnancy.

### Intervention

The LNS, made for the trial by Nutriset, Malaunay, France, contained about 60% calories as fat and 10% calories as protein and came in ready-to-eat packets. Within each treatment arm, the intervention products contained the same daily amounts of vitamins and minerals in both treatment stages (Table 1). Control LNS contained vehicle and flavourings similar to vitamin and mineral-enriched LNS (LNS-VM); it contained micronutrients intrinsic to the bulk ingredients but had no added vitamins or minerals. In the first stage, from recruitment to 2 weeks after starting ART, the products were given with minimal calories, i.e., 30 g/day, approximately 150 kcal/day. From 2 to 6 weeks after initiating ART patients were given 250 g/day, in two 125 g sachets, comprising approximately 1,400 kcal/day. In pilot work at the Mwanza site, the LNS products were found acceptable to a similar group of HIV patients [24]. Products were further evaluated by study staff at both African sites to confirm that the intervention and control preparations were acceptable and indistinguishable.

**Table 1:** **Nutritional composition of trial supplements – amounts per day** ^**a**^

**Nutrient**	**First phase supplement (from recruitment to 2 weeks of ART)**		**Second phase supplement (from 2 to 6 weeks of ART)**	
	**LNS-VM (30 g)**	**LNS (30 g)**	**LNS-VM (250 g)**	**LNS (250 g)**
Calories (kcal)	139	168	1,397	1,416
Protein (g)	2.4	2.3	55	55
Fat (g)	11.0	10.9	97.5	97.5
Potassium (mmol)	30	0.9	32	15.8
Phosphorus (mmol)	47	0.4	38	9.3
Magnesium (mmol)	16	0.3	17	5.7
Calcium (mg)	29.8	5.0	140	115
Iron (mg)	0.4	0.4	14.7	8.4
Zinc (mg)	21	0.2	21	3.8
Copper (mg)	3.6	0.06	3.6	1.2
Manganese (mg)	4.2	–	4.2	–
Iodine (μg)	420	–	420	–
Selenium (μg)	180	–	180	–
Chromium (μg)	75	–	75	–
Retinol (as palmitate) (μg)	1,800	–	1,800	–
Vitamin D (μg)	10	–	10	–
Vitamin E (mg)	45	–	45	–
Vitamin K (μg)	95	–	95	–
Vitamin C (mg)	120	–	120	–
Thiamin (mg)	2.4	–	2.4	–
Riboflavin (mg)	3.3	–	3.3	–
Niacin (mg)	39	–	39	–
Pyridoxine (mg)	3.6	–	3.6	–
Folate (μg)	600	–	600	–
Vitamin B12 (μg)	4.5	–	4.5	–
Pantothenic acid (mg)	9	–	9	–

^a^Where nutrient contents are provided for both LNS and LNS-VM, these are values from analysis by the manufacturer, accounting for inter-batch variability; where values for only LNS-VM are given, these where not assessed in the prepared foods but refer to amounts added, that is, they do not include those intrinsic to the LNS.

ART, Antiretroviral therapy; LNS, Lipid-based nutritional supplement; LNS-VM, LNS with added vitamins and minerals.

The evidence base for micronutrient supplements for adults on ART is extremely limited, therefore, we based levels in the intervention supplement on previous work by the collaborators [8,9,17,25,26], established supplements used for severely malnourished children [7], and recent estimates of requirements for moderately malnourished children [27]. The fundamental basis for the formulation was three times the Recommended Nutrient Intake (RNI) for British women [28], but no iron during the first phase and only the RNI for iron during the second phase.

Stability, safety, and micronutrient levels of the supplements were monitored regularly by the manufacturer, including in sachets kept for 18 months at the study sites, and found to be adequate.

### Randomisation and masking

Randomisation was conducted by the Data Safety and Monitoring Board (DSMB) statistician using computer-generated blocks of 16 and stratified by site. An allocation code (letters A to H) indicating the contents of the supplement packets were known only to Nutriset and the DSMB statistician. A randomisation code linking the allocation code to study ID numbers was held by the DSMB statistician and site-based study pharmacists who did not know the package contents, had no direct contact with patients, and were instructed not to disclose packaging details to the clinical teams. Packages of LNS-VM and LNS, in both small and large dose formats, were delivered by the producer in lots designated by allocation code. Packets were then labelled with the study ID numbers by the clinic pharmacists at the time packets were dispensed. Eligible participants were recruited to sequential IDs (within sites) by clinic nurses with no access to either code. Participants were asked, on exit from the study, if they could guess their treatment. Only 616/1,256 (49%) patients said they could guess, and of these, 83% in both treatment groups guessed they were on the high vitamins and minerals supplement.

### Adherence

At each visit, patients were provided with sufficient sachets of LNS or LNS-VM to last until their next scheduled visit. Adherence to both intervention supplements was monitored by asking participants to return empty packages at their next visit. Overall adherence was calculated as total empty packages returned divided by total packages expected to be consumed. When patients died or stopped attending study visits, data on returned packages was missing. In these cases, it was assumed that all of the packages given at their last study visit were consumed. A sensitivity analysis was carried out assuming that none of these packages were consumed.

### Sample size justification

The primary outcome was mortality within the study period which was estimated to include about 4 weeks pre-ART and 12 weeks of ART. Calculations of sample size were based on two studies from urban Africa: one study in Cape Town [29], which separated the high mortality pre-ART phase from mortality after starting ART, and one from Lusaka, with a patient population similar to that expected in the proposed study [3]. We assumed a mortality rate in the control group of 25/100 person-years, based on post-ART mortality of Zambian patients starting ART with a BMI of 17 to 18.49 kg/m^2^. We calculated that 1,150 patients per treatment arm, with 5% loss to follow-up, would provide >90% power to detect a 50% reduction in mortality, similar to the mortality difference between Zambian patients with plasma phosphate above and below the median [8], and less than the mortality reduction achieved through multiple micronutrient supplementation of Thai patients with low CD4 counts before ART was locally available [25]. It became apparent, by June 2013, that the trial would be unable to recruit all planned participants within the funding available; however, mortality was higher than expected (see Results). The steering group asked the DSMB to consider futility, in addition to a planned interim efficacy analysis, at this point. The DSMB recommended that recruitment could cease. Therefore, recruitment stopped in July 2013 with a total of 1,815 participants which was estimated, considering actual mortality, as sufficient to detect, with 90% power, a reduction in mortality of 30%.

### Participant recruitment and follow-up

Adults attending free HIV testing services at both sites, eligible for ART and fulfilling the study inclusion criteria, were deemed eligible for the trial. Before initiating ART as part of routine care, patients were screened, commenced on treatment for opportunistic infections, and counselled regarding lifelong treatment adherence. During this pre-ART period, the first stage study interventions were introduced. Medical care was provided primarily by local health services, although study staff treated and referred as necessary during the patients’ study visits. National treatment guidelines for ART regimen differed in the two countries: most Zambians were prescribed Efavirenz/Tenofovir/Emtricitabine, whereas a wider range of regimens were prescribed to Tanzanians.

Patients were seen weekly from recruitment until the ART initiation visit, then at 2, 4, 6, 8, and 12 weeks after starting ART. Patients who were ill could come for unscheduled visits at any time. Patients were actively followed-up if they missed scheduled visits; patients and relatives were phoned, their ART clinics were contacted, and, in Mwanza, they were visited at home. We were thus able to ascertain the primary outcome, mortality, on a greater number of patients than those attending the week 12 visit for secondary outcomes.

### Outcomes

The primary outcome was death between recruitment and 12 weeks after starting ART, based either on reports from medical facilities or from relatives. In order to capture serious illnesses which did not result in death, a second outcome was hospitalisation, defined as at least overnight admission to a hospital, including inpatient facilities attached to clinics. Diagnoses and duration of hospitalisation were recorded.

SAEs comprised a combination of events that resulted in death, hospitalisation, or permanent disability, or were life-threatening, as well as a drug overdose or cancer. Low serum phosphate and low or high serum potassium levels of US National Institutes of Health Division of AIDS (DAIDS [30]) grade 3 and 4 were considered severe laboratory adverse events. DAIDS does not set high ranges for phosphate but, because we were also interested in potential excesses from supplementation, we classified any above-normal limits as adverse events [31]. Analysis of magnesium levels was also planned, however for technical reasons, results were only available for few patients and did not show much of clinical interest or association with other data, and were therefore omitted. Alanine aminotransferase (ALT) at baseline and 12 weeks after starting ART was investigated in a non-random subset of patients recruited later in the trial following a report of raised ALT associated with high dose micronutrients in a trial of HIV patients in Tanzania [32].

Weight was measured at all visits and height at recruitment if the patient was able to stand; data from the screening visit were used if the patient was unable to stand. Measurements were taken in triplicate and the median used in analyses. Data for the final visit were used for all who attended up to 14 days before or after the official follow-up end date of 12 weeks since starting ART.

### Laboratory analyses

Venous blood samples were taken at all scheduled visits. CD4 count (baseline and week 12 only) was measured by local central clinical services. In Lusaka, serum phosphate was measured spectrochemically on a Pointe 180 analyser (Bactlabs East Africa, Nairobi, Kenya). Sample results were accepted only from runs for which the external quality control (QC) sample from the same supplier was within expected range. Inter-assay coefficient of variation (CV) for this external QC was 7%. In Mwanza, serum phosphate was measured in an external laboratory (Bugando Medical Centre) using a Roche COBAS Integra 400 analyser. Serum potassium was measured by optical emission using Perkin Elmer Optima 7000 ICP (Perkin Elmer, South Africa). An external QC (Seronorm, Alere, Cheshire, UK) was run each day and values were within expected limits at both sites. CVs for potassium were 5% in Mwanza and 6% in Lusaka. Both sites experienced periods when the Optima machines were out of order so samples were sent to Bugando laboratory in Mwanza and the Centre for Infectious Diseases Research in Zambia (CIDRZ) laboratory in Lusaka. The Pointe 180 was used at both sites to measure ALT. Values for the external QCs (Bactlabs East Africa, Nairobi, Kenya) were very slightly under the expected ranges at both sites; inter-assay CVs were 14% in Lusaka and 10% in Mwanza.

### Data management and statistics

Data were double entered into OpenClinica data management system in Lusaka and into CSPro 4.1 and stored in MySQL databases in Mwanza. Analyses were conducted in STATA version 13.1. Primary statistical analysis was by intention-to-treat, and per protocol analyses were also conducted to account for adherence to treatments. The primary analysis compared mortality rates between treatment groups by Cox regression. Robust standard errors Cox regression was performed to determine effects of the intervention on hospitalisation rate and occurrence of clinical and laboratory SAEs accounting for repeat events. Proportional hazards assumptions were examined using cumulative hazard plots and Schoenfeld residuals [33]. Mean BMI and CD4 at 12 weeks were compared between groups using *t*-tests and linear regression, adjusting for baseline values. Pre-planned analyses determined whether treatment effects were modified by country, sex, initial BMI (< or ≥17 kg/m^2^), initial CD4 count (< or ≥100 cells/μL), or TB treatment pre-ART using Cox regression with an interaction term between treatment arm and potential effect modifier. Principal components analysis, used to describe the socio-economic status of the population [34], was conducted separately for each country since there were notable differences between sites; variables offered into the principal components analysis were housing characteristics, sanitation, water source, and ownership of electrical goods, animals, and modes of transport.

### Ethics

Ethical approval was obtained from the research ethics committee of the London School of Hygiene and Tropical Medicine, the University of Zambia Biomedical Research Ethics Committee, and the Medical Research Coordinating Committee of National Institute for Medical Research, Tanzania. All participants provided written or thumbprint informed consent. Medical care of patients was according to national guidelines and provided through the local health services. Patients with low levels of serum electrolytes according to DAIDS criteria were provided appropriate electrolyte therapy.

## Results

### Participants and follow-up

**Figure 1:**
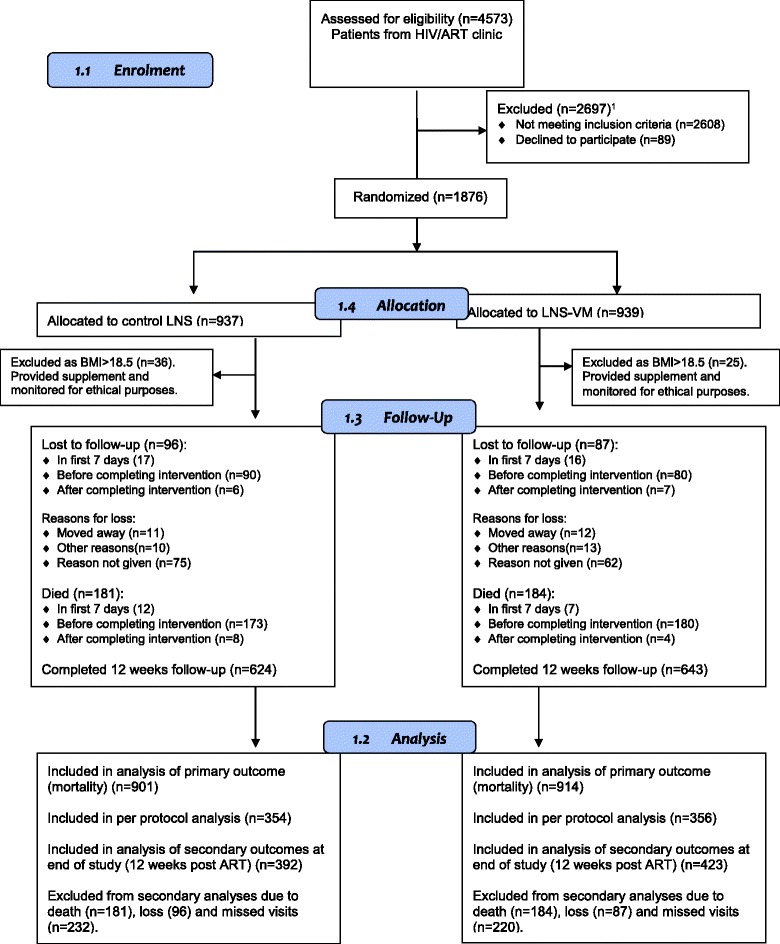
**Flow of participants through the study.** Screening in Mwanza was of all HIV-infected patients referred for CD4 testing, whereas in Lusaka only patients who also had body mass index <18.5 kg/m^2^ were formally screened; this resulted in a greater proportion of ineligible patients in Mwanza. LNS, Lipid-based nutritional supplement without added vitamins and minerals; LNS-VM, Lipid nutritional supplement with added vitamins and minerals. ^1^ Not meeting inclusion criteria (n = 2,608): 5 < 18 yr, 17 non-ART naive, 303 BMI >18.5 kg/m^2^, 16 unwilling for intensive follow-up, 10 pregnant, 4 enrolled in other study, 21 refused CD4 count, 2,222 not eligible for ART, 10 unwilling to start ART.

Figure 1 shows the flow of participants through the study and Table 2 describes the population at recruitment. Characteristics of the population were similar at baseline between treatment arms. Compared with Zambian participants, Tanzanian participants had lower CD4 counts and blood haemoglobin, had less education, and were more likely to be self-employed but less likely to be salaried or unemployed (data not shown). Oedema was uncommon at baseline and all patients with oedema had BMI <18.5 kg/m^2^ at recruitment. Mean CD4 count of participants was 137 cells/μL (SD 100). Low plasma phosphate was found in 196 patients (11%), low potassium in 274 (16%), and anaemia was common at recruitment. A quarter of participants were started on TB treatment before initiating ART.

**Table 2:** **Baseline characteristics of the study population**

**Variable**	**Level**	**LNS-VM**	**LNS**
n (%)		914 (50.4)	901 (49.6)
Age (years), mean (SD)		35.9 (9.4)	35.7 (9.4)
Female, n (%)		443 (49)	457 (51)
Marital status, n (%)	Married/Cohabiting	420 (46)	438 (49)
	Widowed	96 (11)	107 (12)
	Divorced/Separated	266 (29)	244 (27)
	Single	132 (14)	111 (12)
	Missing	0 (0)	1 (0.1)
Occupation, n (%)	Salaried	134 (15)	137 (15)
	Self-employed	485 (53)	460 (51)
	Housewife	86 (9)	97 (11)
	Student	9 (1)	9 (1)
	Unemployed	200 (22)	197 (22)
	Missing	0 (0)	1 (0.1)
Education, n (%)	None	170 (19)	173 (19)
	Primary	528 (58)	517 (57)
	Secondary	192 (21)	185 (21)
	Tertiary	24 (3)	25 (3)
	Missing	0 (0)	1 (0.1)
Socioeconomic quintiles^b^, n (%)	Lowest	166 (18)	198 (22)
	Low	185 (20)	189 (21)
	Middle	200 (22)	162 (18)
	High	173 (19)	182 (20)
	Highest	190 (21)	170 (19)
BMI (kg/m^2^), mean (sd)		16.4 (1.4)	16.4 (1.4)
BMI <17 kg/m^2^, n (%)		542 (59)	532 (59)
CD4 count (cells/μL), mean (SD)		134 (97)	139 (103)
CD4 count group, n (%) at each	<50	227 (25)	216 (24)
range of cells/μL	50–99	171 (19)	176 (20)
	100–199	279 (31)	252 (28)
	≥200	237 (26)	257 (29)
Haemoglobin (g/L), mean (SD)		95 (23)	97 (24)
Haemoglobin group^a^, n (%)	Severe anaemia	207 (23)	191 (21)
	Moderate anaemia	422 (46)	388 (43)
	Mild anaemia	128 (14)	157 (17)
	Normal	82 (9)	95 (11)
	Missing	75 (8)	70 (8)
Phosphate <0.87 mmol/L, n (%)	<0.87	113 (12)	83 (9)
	Missing	26 (3)	25 (3)
Potassium <3.5 mmol/L, n (%)	<3.5	133 (15)	141 (16)
	Missing	53 (6)	52 (6)
TB treatment pre-ART, n (%)		252 (28)	199 (22)
Oedema at baseline, n (%)		31 (3)	35 (4)
Initial ART regimen, n (%)	AZT/3TC/EFV	68 (10)	67 (9)
	AZT/3TC/NVP	111 (16)	125 (17)
	TDF/FTC/EFV	418 (58)	398 (55)
	TDF/FTC/NVP	31 (4)	33 (5)
	Other	30 (4)	24 (3)
	Missing	59 (8)	71 (10)

BMI, Body mass index; CI, Confidence interval; LNS, Lipid-based nutritional supplement without added vitamins and minerals; LNS-VM, Lipid nutritional supplement with added vitamins and minerals; AZT, Zidovudine; 3TC, Lamivudine; NVP, Nevirapine; TDF, Tenofovir; EFV, Efavirenz; FTC, Emtricitabine.

^a^Haemoglobin categories were selected based on usual nutritional cut-offs, not as defined for specific adverse events; adequate haemoglobin was defined as 130 g/L for men and 120 g/L for women; mild anaemia was 110 g/L to the adequate cut-off; moderate anaemia was 80–109 g/L; severe anaemia was <80 g/L.

^b^Socioeconomic quintiles analysed for each site separately by principal component analysis.

Total follow-up time was 439 person-years. Participants attended 6,657 clinic visits in the LNS-VM arm and 6,282 clinic visits in the LNS arm over the course of the study. The median time between study enrolment and ART initiation was 21 days (interquartile range (IQR), 15–30) in both the LNS-VM and the LNS arm. Overall median follow-up periods were: all participants, 14.3 weeks (IQR, 10.4–15.7); all those who completed follow-up, 15 weeks (IQR, 14.1–16.3); all those who died, 5.7 weeks (IQR, 3.1–9.1); and all those lost to follow-up, 3.1 weeks (IQR, 1–8.1). There were 173 (9.5%) participants who were lost to study follow-up or withdrew consent and were censored at last contact with study team; median duration of follow-up did not differ between treatment arms.

### Mortality

**Figure 2:**
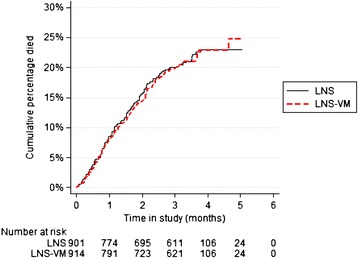
**Effect of treatment allocation on mortality.** LNS, Lipid-based nutritional supplement without added vitamins and minerals; LNS-VM, Lipid nutritional supplement with added vitamins and minerals. The X axis presents total time in the study in months, irrespective of individual times after recruitment when a patient started antiretroviral therapy (ART); median time between recruitment and start of ART was 21 days (0.75 months).

There were 184 deaths in the LNS-VM arm, 82.6/100 person-years (95% CI, 71.4–95.4), and 181 deaths in the LNS arm, 83.7/100 person-years (95% CI, 72.3–96.8; Figure 2 and Table 3). The mortality rate ratio (RR) was 0.99 (95% CI, 0.80–1.21; *P* = 0.89). In planned stratified regression analyses, participant country, sex, initial BMI, initial CD4 count, initial low phosphate or potassium, or whether they were on TB treatment before starting ART did not modify the effect of the intervention on mortality. There was also no effect of the intervention on mortality if the pre- and post-starting ART periods were examined separately.

**Table 3:** **Effects of intervention on overall and subgroup mortality**

		**n**	**Deaths**	**Mortality rate per 100 person-years (95% CI)**	**Rate ratio** ^**b**^ **(95% CI)**	***P*** **value**	***P*** **value for modification**
Total intention to treat analysis	LNS-VM	914	184	82.6 (71.4–95.4)	0.99 (0.80–1.21)	0.89	
	LNS	901	181	83.7 (72.3–96.8)			
Per protocol analysis^a^	LNS-VM	356	56	64.2 (49.4–83.4)	0.84 (0.59–1.21)	0.35	
	LNS	354	64	76.9 (60.2–98.2)			
Pre-ART period	LNS-VM	896	79	113.6 (91.2–141.7)	1.0 (0.75–1.43)	0.81	
	LNS	888	72	108.0 (85.7–136.0)			0.53
Post-ART period	LNS-VM	740	97	64.1 (52.5–78.2)	0.91 (0.69–1.19)	0.49	
	LNS	741	105	70.8 (58.5–85.7)			
Men	LNS-VM	471	105	94.9 (78.4 to114.9)	0.97 (0.74–1.27)	0.82	
	LNS	444	104	97.5 (80.4–118.2)			0.87
Women	LNS-VM	443	79	70.4 (56.4–87.7)	1.00 (0.73–1.37)	0.98	
	LNS	457	77	70.2 (56.2–87.8)			
Zambia	LNS-VM	559	85	60.8 (49.1–75.2)	0.96 (0.71–1.30)	0.80	
	LNS	552	85	63.2 (51.1–78.2)			0.81
Tanzania	LNS-VM	355	99	119.3 (97.9–145.2)	1.01 (0.76–1.34)	0.93	
	LNS	349	96	117.3 (96.0–143.3)			
BMI <17.0 kg/m^2^	LNS-VM	542	135	106.8 (90.2–126.4)	1.05 (0.83–1.34)	0.68	
	LNS	532	124	101.4 (85.0–120.9)			0.32
BMI ≥17.0 kg/m^2^	LNS-VM	372	49	50.8 (38.4–67.2)	0.84 (0.57–1.22)	0.36	
	LNS	369	57	60.6 (46.8–78.6)			
CD4 < 100 /μL	LNS-VM	398	107	117.3 (97.0–141.8)	0.89 (0.69–1.16)	0.39	
	LNS	392	117	131.7 (109.9–157.9)			0.22
CD4 ≥ 100 /μL	LNS-VM	516	77	58.5 (46.8–73.1)	1.16 (0.83–1.62)	0.38	
	LNS	509	64	50.2 (39.3–64.1)			
On TB treatment before ART	LNS-VM	252	38	57.9 (42.2–79.6)	1.30 (0.78–2.16)	0.32	
	LNS	199	24	44.3 (29.7–66.1)			0.29
Not on TB treatment before ART	LNS-VM	662	146	92.8 (78.9–109.2)	0.96 (0.76–1.20)	0.71	
	LNS	702	157	96.8 (82.8–113.2)			
Phosphate <0.87 mmol/L	LNS-VM	113	25	93.5 (63.1–138.3)	0.99 (0.54–1.79)	0.96	
	LNS	83	19	93.5 (59.6–146.5)			0.998
Phosphate ≥0.87 mmol/L	LNS-VM	775	152	79.8 (68.0–93.5)	0.99 (0.79–1.23)	0.90	
	LNS	793	154	80.9 (69.1–94.7)			
Potassium <3.5 mmol/L	LNS-VM	133	34	113.8 (81.3–159.3)	0.87 (0.55–1.37)	0.54	
	LNS	141	40	130.3 (95.6–177.7)			0.69
Potassium ≥3.5 mmol/L	LNS-VM	728	137	76.0 (64.3–89.8)	0.96 (0.76–1.22)	0.76	
	LNS	708	136	79.3 (67.1–93.8)			

BMI, Body mass index; CI, Confidence interval; LNS, Lipid-based nutritional supplement without added vitamins and minerals; LNS-VM, Lipid nutritional supplement with added vitamins and minerals; TB, Tuberculosis.

^a^Included only participants who consumed at least 75% of their expected supplement sachets; ^b^Rate ratio from Cox regression analysis.

Only 710 (39%) of patients consumed at least 75% of the expected number of sachets of supplement for their time in the study. When the mortality analysis was restricted to these patients with higher adherence to the intervention, mortality rates were lower than in the full cohort but there remained no evidence of a difference in RR (RR, 0.84; 95% CI, 0.59–1.21). In the sensitivity analysis, assuming patients did not consume supplements from their last visit, the RR was 1.19 (95% CI, 0.61–2.35). Adherence to ART medication was very good: adherence of at least 95% was observed in 622 (96%) of those receiving LNS-VM and in 607 (95%) of those receiving LNS (RR, 1.1; 95% CI, 0.64–1.88, *P* = 0.73).

### Secondary outcomes

**Figure 3:**
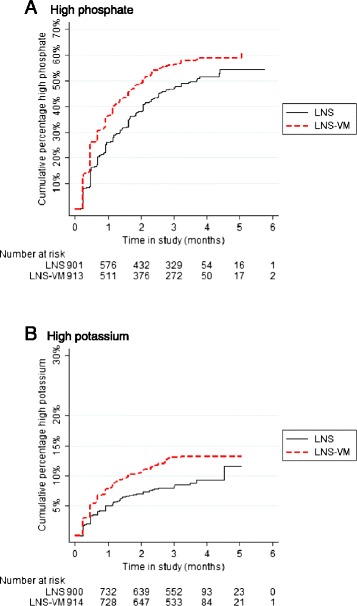
**Effect of treatment allocation on time to first event of plasma phosphate or potassium higher than the normal range.** The cut-offs for values greater than the normal range were 1.45 mmol/L for phosphate and 5.5 mmol/L for potassium [31]. LNS, Lipid-based nutritional supplement without added vitamins and minerals; LNS-VM, Lipid nutritional supplement with added vitamins and minerals.

There was no evidence of differences between treatment arms in clinical SAEs, although there was a trend towards lower rates in the LNS-VM group (Table 4). There was evidence of a decreased incidence of severely low phosphate in the LNS-VM group. Examining all levels above the normal range, there was strong evidence of increased incidence of both high phosphate and high potassium in the LNS-VM group (Figure 3).

**Table 4:** **Effect of intervention on adverse events (AEs)**

		**Events**	**Event rate per 100 person-years (95% CI)**	**Rate ratio (95% CI)** ^**a**^	***P*** **value**
**Clinical AEs**					
Hospitalisation	LNS-VM	170	76.3 (65.6–88.6)	0.87 (0.71–1.07)	0.19
	LNS	190	87.8 (76.2–101.2)		
All serious clinical adverse events^b^	LNS-VM	250	112.2 (99.1–127.0)	0.89 (0.75–1.06)	0.20
	LNS	272	125.7 (111.6–141.6)		
**Severe laboratory AEs** ^**c**^					
Potassium >6.5 mmol/L	LNS-VM	29	13.0 (9.0–18.7)	1.66 (0.91–3.02)	0.097
	LNS	17	7.9 (4.9–12.6)		
Potassium <2.5 mmol/L	LNS-VM	28	12.6 (8.7–18.2)	0.82 (0.50–1.36)	0.44
	LNS	33	15.3 (10.8–21.5)		
Phosphate <0.65 mmol/L	LNS-VM	86	38.7 (31.2–47.7)	0.73 (0.55–0.97)	0.03
	LNS	114	52.7 (43.9–63.3)		
**Abnormally high electrolyte levels** ^**d**^					
Potassium >5.5 mmol/L	LNS-VM	117	52.5 (43.8–62.9)	1.60 (1.19–2.15)	0.002
	LNS	71	32.8 (26.0–41.4)		
Phosphate >1.45 mmol/L	LNS-VM	650	219.6 (270.1–314.9)	1.23 (1.10–1.37)	<0.001
	LNS	513	237.1 (217.5–258.6)		

CI, Confidence interval; LNS, Lipid-based nutritional supplement without added vitamins and minerals; LNS-VM, Lipid nutritional supplement with added vitamins and minerals.

^a^Accounting for repeat events by patient; ^b^Death, hospitalisation, life-threatening events, permanently disabling, congenital abnormality, cancer, drug overdose; ^c^Plasma potassium or phosphate levels meeting DAIDS criteria severity 3 or 4; ^d^DAIDS criteria severity at least 1 for potassium and above normal [31] for phosphate for which DAIDS has no high level cut-offs.

After controlling for baseline CD4 count, there was evidence that mean CD4 count at 12 weeks post-ART was higher in the LNS-VM compared with the LNS group (adjusted difference, 25 cells/μL; 95% CI, 4–46; *P* = 0.02; Table 5). Controlling for baseline BMI, mean BMI at 12 weeks was not significantly greater in the LNS-VM compared with the LNS group. There was no evidence that mildly elevated ALT (>40 U/L) at 12 weeks was more prevalent in the LNS-VM group (12/74 (16%) versus 6/62 (10%) in the LNS group; *P* = 0.26).

**Table 5:** **Effects of intervention on body mass index (BMI), blood CD4 count, and alanine aminotransferase (ALT)**

		**n**	**Mean at end of study (SD)**	**Difference in means (95% CI)**	***P*** **value**	**Adjusted difference in means** ^**a**^ **(95% CI)**	***P*** **value**
BMI (kg/m^2^)	LNS-VM	421	18.5 (1.8)	0.15 (−0.10–0.41)	0.23	0.15 (−0.08–0.37)	0.20
	LNS	392	18.4 (1.9)				
CD4 count/μL	LNS-VM	404	297 (188)	17 (−8–42)	0.18	25 (4–46)	0.02
	LNS	355	280 (154)				
ALT (U/L)	LNS-VM	74	26.4 (1.4)	−0.3 (−4.4–3.9)	0.90	−0.98 (−5.2–3.3)	0.65
	LNS	62	26.6 (1.6)				

CI, Confidence interval; LNS, Lipid-based nutritional supplement without added vitamins and minerals; LNS-VM, Lipid nutritional supplement with added vitamins and minerals.

^a^Adjusted for baseline values; sample size for adjusted analysis for ALT was reduced because of missing baseline values: 71 in LNS-VM, 61 in LNS.

## Discussion

High early mortality in ART programmes in sub-Saharan Africa is an ongoing concern, and early work suggested that nutritional interventions may hold out some hope of benefit. In this randomised controlled trial of a fairly high dose, usually three times the RNI, of vitamins and minerals, added to a two-stage lipid-based nutritional supplement, we found no reduction in mortality in the intervention group. A trial comparing very high doses of vitamins with one RNI in a similar population also found no benefits for mortality [32]. The NUSTART nutritional intervention did, however, have a modest benefit for CD4 count.

The trial benefited from its large sample size, excellent follow-up for the primary outcome, and regular collection of detailed clinical and laboratory data. A limitation was the need to stop recruitment before the originally planned number of patients; although this did not jeopardise the primary outcome because of the unexpectedly high mortality rate, it may nevertheless have limited power to detect changes in some secondary outcomes. A second limitation was the low proportion of people with adequate compliance to the intervention. Because our pilot data showed the supplements were well liked [24], we think low compliance is more likely due to the difficulties of following up very ill patients for long-term care in urban and periurban African environments – patients needed to attend study visits both to collect supplements and to return empty sachets for compliance estimation – as well as the greater difficulties for patients with regards to compliance with high calorie food interventions than with pills. Compliance could have been overestimated if patients shared their sachets with household members. However, we think this unlikely as the importance of taking the supplement was emphasised at each study visit, patients were questioned at each visit, and sharing was reported in less than 1% of cases. Furthermore, compliance could have been underestimated as we relied on patients returning their empty sachets. Finally, although we based the amount of LNS supplied on amounts used in similar studies [14,16], the full amount may not be required to improve CD4 count and some anthropometric measures (Rehman et al., submitted), since benefits were seen despite poor adherence. Future studies of LNS provision could perhaps save costs by providing smaller amounts.

The NUSTART intervention was based on similar nutritional supplementation strategies used for severely malnourished young children and includes several underlying concepts: electrolyte metabolism and requirement for tissue deposition; micronutrient metabolism, especially for iron and anti-oxidants; and the need to stabilise metabolism before giving high calorie supplements to promote nutritional recovery. Our results suggest that these underlying concepts may have had a different importance for the health of our patient population, with consequent mixed outcomes.

First, regarding electrolyte metabolism and requirements, our results suggest that, although low levels of phosphate were a risk factor for death in a similar group of Zambian patients previously reported [8], the hypothesis that providing supplemental oral electrolytes would decrease mortality risk was not proved correct. It is possible that any benefits of supplementation were masked by the intensive medical care provided by the trial clinical teams. We measured patient blood electrolytes at all visits in order to inform our understanding of the effect of the intervention and for patient safety. Patients with low electrolytes were found slightly more often in the LNS than the LNS-VM group. For ethical reasons, these patients were treated (whenever they could be traced quickly, which was not always possible in the context of sprawling African urban and periurban areas) usually with oral supplements. We do not know whether this treatment prevented some severe derangements and thus hospitalisations and deaths.

We did not expect to find many cases of high electrolytes with the supplement as designed and were surprised by the increased rate of both phosphate and potassium events. The amount of potassium (~30 mmol/day) in the LNS-VM was actually low in terms of potassium balance in a healthy adult (RNI 50 to 90 mmol/day [28]), but was the maximum the manufacturer was able to add without compromising taste and acceptability of the supplement. However, in some participants, the amount appeared to be more than their metabolism could handle. The daily dose of phosphate was almost triple the RNI since we have previously shown that low phosphate is associated with a poor outcome in this patient group [8]. Not all of the adverse electrolyte events recorded would be clinically significant; for example, short term rises in plasma phosphate do not predictably lead to any clinical consequences. High potassium is more worrying and is the only one of the electrolytes we measured which has DAIDS criteria listed for high values. The evidence for a difference in severely high potassium is weak, but this could be a result of the small numbers involved. There were 175 people who developed a potassium value above the normal range of whom 24 (14%) died. While this is a lower risk of death than for the whole study cohort, it is not a reason for complacency as the longer a patient stayed alive in the study, the more opportunity they had to be diagnosed with an abnormal electrolyte value. Further, among these 175 patients, the mortality rate was two and half times higher in those taking LNS-VM (hazards ratio, 2.54; 95% CI, 0.95–6.82; *P* = 0.06) suggesting their electrolyte abnormality was more severe. This is an important finding and we would recommend that malnourished HIV-positive people are not given blanket treatment with potassium supplements. The addition of phosphate to nutrition supplements should be kept at low levels.

The second underlying concept for the supplement and trial design – that high levels of electrolytes and micronutrients would promote recovery – is supported by the modest increase in CD4 count in the LNS-VM group. A recent review found few nutritional supplementation trials with CD4 count as an outcome and no evidence of benefits of food or nutrition education interventions [13]. A review focussing on micronutrient supplements in HIV found some evidence of benefits of multiple micronutrient supplements for CD4 count [18]; however, evidence was limited and much of the research focussed on pregnant women or was conducted before ART was widely available. Thus, vitamin and mineral supplementation to improve immunological recovery in seriously ill HIV patients clearly merits further investigation, especially since early robust recovery of CD4 count may be particularly important for survival of patients with a low BMI [35]. Other aspects of nutritional recovery are among the NUSTART trial secondary outcomes to be reported later. It should be noted that, because patients were severely malnourished, the control group received LNS, which contained innate vitamins and minerals as well as calories and may have provided health benefits; this is supported by a trial of less malnourished patients in Ethiopia in which inclusion of a delayed supplementation control group demonstrated nutritional benefits of providing LNS at the start of ART [16].

The mortality rate seen in our trial was higher than reported in most studies for patients starting ART, including in the two studies used to estimate the required sample size [3,29]. We believe this was due to a combination of the lower BMI of patients in NUSTART than in the other cohorts and on our inclusion of patients during the high-risk period immediately pre-ART [29]. We note that NUSTART post-ART initiation mortality rates were similar to rates for the same BMI categories in another study from Tanzania [4], but we are unaware of other studies that have reported outcomes for this combination of the risk factors of low BMI and the pre-ART interval. We are in the process of analysing the mortality risk-factors in more detail. However, the high mortality seen emphasises the importance of prompt diagnosis and treatment for malnourished HIV-infected people.

We measured serum ALT in only a subset of patients after a trial with a high micronutrient supplement for a similar group of HIV-infected patients found increased ALT in the intervention group [32]. However, we found no differences in ALT between treatment arms, suggesting that this may not be a serious issue.

## Conclusions

In summary, the intervention protocol did not decrease mortality or SAEs, although it did benefit a secondary outcome, CD4 count. It is possible that the electrolyte component of the supplement had no benefits due to the study design, where electrolytes were measured regularly and electrolyte therapy provided as needed, and to the excess of high electrolytes. Nevertheless, the benefit for CD4 count in this large trial, added to previous information from smaller trials of micronutrient supplements for HIV patients, suggests that micronutrient supplementation should be pursued. Further research into metabolic causes of the early mortality among malnourished Africans starting ART is warranted. It will also be important to address how to improve compliance with similar nutritional interventions. Finally, it will be informative to follow-up the NUSTART patients to determine whether modest early changes in CD4 count or nutritional status have long-term effects.

## Abbreviations

ALT: Alanine aminotransferase
ART: Antiretroviral therapy
BMI: Body mass index
CV: Coefficient of variation
DAIDS: Division of AIDS
DSMB: Data Safety and Monitoring Board
IQR: Interquartile range
LNS: Lipid-based nutritional supplements
LNS-VM: Vitamin and mineral supplements in a lipid-based nutritional supplement
NUSTART: Nutritional Support for Adults Starting Antiretroviral Therapy
QC: Quality control
RNI: Recommended Nutrient Intake
RR: Rate ratio
SAEs: Serious adverse events
TB: Tuberculosis

## References

[1] Lawn SD, Harries AD, Anglaret X, Myer L, Wood R (2008). Early mortality among adults accessing antiretroviral treatment programmes in sub-Saharan Africa. AIDS..

[2] Stringer JS, Zulu I, Levy J, Stringer EM, Mwango A, Chi BH, Mtonga V, Reid S, Cantrell RA, Bulterys M, Saag MS, Marlink RG, Mwainga A, Ellerbrock TV, Sinkala M (2006). Rapid scale-up of antiretroviral therapy at primary care sites in Zambia: feasibility and early outcomes. JAMA..

[3] Koethe JR, Lukusa A, Giganti MJ, Chi BH, Nyirenda CK, Limbada MI, Banda Y, Stringer JS (2010). Association between weight gain and clinical outcomes among malnourished adults initiating antiretroviral therapy in Lusaka. Zambia. J Acquir Immune Defic Syndr..

[4] Liu E, Spiegelman D, Semu H, Hawkins C, Chalamilla G, Aveika A, Nyamsangia S, Mehta S, Mtasiwa D, Fawzi W (2011). Nutritional status and mortality among HIV-infected patients receiving antiretroviral therapy in Tanzania. J Infect Dis..

[5] Hsu J, Pencharz P, Macallan D, Tomkins A (2005). Macronutrients and HIV/AIDS: A Review of Current Evidence.

[6] Macallan DC, Noble C, Baldwin C, Foskett M, McManus T, Griffin GE (1993). Prospective analysis of patterns of weight change in stage IV human immunodeficiency virus infection. Am J Clin Nutr..

[7] Ashworth A, Khanum S, Jackson A, Schofield C (2003). Guidelines for the Inpatient Treatment of Severely Malnourished Children.

[8] Heimburger DC, Koethe JR, Nyirenda C, Bosire C, Chiasera JM, Blevins M, Munoz AJ, Shepherd BE, Potter D, Zulu I, Chisembele-Taylor A, Chi BH, Stringer JS, Kabagambe EK (2010). Serum phosphate predicts early mortality in adults starting antiretroviral therapy in Lusaka, Zambia: a prospective cohort study. PLoS One..

[9] Koethe JR, Blevins M, Nyirenda C, Kabagambe EK, Shepherd BE, Wester CW, Zulu I, Chiasera JM, Mulenga LB, Mwango A, Heimburger DC (2011). Nutrition and inflammation serum biomarkers are associated with 12-week mortality among malnourished adults initiating antiretroviral therapy in Zambia. J Int AIDS Soc..

[10] Prentice A (2008). Iron metabolism, malaria and other infections: what is all the fuss about?. J Nutr..

[11] Collins S, Myatt M, Golden B (1998). Dietary treatment of severe malnutrition in adults. Am J Clin Nutr..

[12] Academy of Science of South Africa (2007). HIV/AIDS, TB and Nutrition.

[13] Grobler L, Siegfried N, Visser ME, Mahlungulu SS, Volmink J (2013). Nutritional interventions for reducing morbidity and mortality in people with HIV. Cochrane Database Syst Rev..

[14] Ndekha MJ, van Oosterhout JJ, Zijlstra EE, Manary M, Saloojee H, Manary MJ (2009). Supplementary feeding with either ready-to-use fortified spread or corn-soy blend in wasted adults starting antiretroviral therapy in Malawi: randomised, investigator blinded, controlled trial. BMJ..

[15] Ndekha M, van Oosterhout JJ, Saloojee H, Pettifor J, Manary M (2009). Nutritional status of Malawian adults on antiretroviral therapy 1 year after supplementary feeding in the first 3 months of therapy. Trop Med Int Health..

[16] Olsen MFAA, Kæstel P, Tesfaye M, Yilma D, Girma T, Wells JCK, Ritz C, Mølgaard C, Michaelsen KF, Zerfu D, Brage S, Andersen AB, Friis H (2014). Effects of nutritional supplementation for HIV patients starting antiretroviral treatment: randomised controlled trial in Ethiopia. BMJ..

[17] Kelly P, Katubulushi M, Todd J, Banda R, Yambayamba V, Fwoloshi M, Zulu I, Kafwembe E, Yavwa F, Sanderson IR, Tomkins A (2008). Micronutrient supplementation has limited effects on intestinal infectious disease and mortality in a Zambian population of mixed HIV status: a cluster randomized trial. Am J Clin Nutr..

[18] Irlam JH, Visser MM, Rollins NN, Siegfried N (2010). Micronutrient supplementation in children and adults with HIV infection. Cochrane Database Syst Rev..

[19] Gibson R, Kafwembe E, Mwanza S, Gosset L, Bailey K, Mullen A, Baisley K, Filteau S (2011). A micronutrient-fortified food enhances iron and selenium status of Zambian infants but has limited efficacy on zinc. J Nutr..

[20] Gitau R, Makasa M, Kasonka L, Sinkala M, Chintu C, Tomkins A, Filteau S (2005). Maternal micronutrient status and decreased growth of Zambian infants born during and after the maize price increases resulting from the Southern African drought of 2001–2002. Pub Health Nutr..

[21] PrayGod G, Range N, Faurholt-Jepsen D, Jeremiah K, Faurholt-Jepsen M, Aabye MG, Jensen L, Jensen AV, Grewal HM, Magnussen P, Changalucha J, Andersen AB, Friss H (2011). Daily multi-micronutrient supplementation during tuberculosis treatment increases weight and grip strength among HIV-uninfected but not HIV-infected patients in Mwanza. Tanzania. J Nutr..

[22] Marston M, Michael D, Wringe A, Isingo R, Clark BD, Jonas A, Mngara J, Kalongoji S, Mbaga J, Changalucha J, Todd J, Zaba B, Urassa M (2012). The impact of antiretroviral therapy on adult mortality in rural Tanzania. Trop Med Int Health..

[23] Zambian Central Statistical Office, Tropical Diseases Research Centre, University of Zambia, Macro International Inc. (2009). Zambia Demographic and Health Survey 2007.

[24] Hebie M, Jungjohann S, Praygod G, Filteau S (2013). Acceptability of different lipid-based nutrient supplements for adults with HIV. Afr J Food Agricul Nutr Devel.

[25] Jiamton S, Pepin J, Suttent R, Filteau S, Mahakkanukrauh B, Hanshaoworakul W, Chaisilwattana P, Suthipinittharm P, Shetty P, Jaffar S (2003). A randomized trial of the impact of multiple micronutrient supplementation on mortality among HIV-infected individuals living in Bangkok. AIDS..

[26] Friis H (2006). Micronutrient interventions and HIV infection: a review of current evidence. Trop Med Int Health..

[27] Golden M (2009). Proposed nutrient requirements of moderately malnourished populations of children. Food Nutr Bull..

[28] UK Department of Health (1991). Dietary Reference Values for Food Energy and Nutrients for the UK.

[29] Lawn SD, Little F, Bekker LG, Kaplan R, Campbel E, Orrell C, Wood R (2009). Changing mortality risk associated with CD4 cell response to antiretroviral therapy in South Africa. AIDS..

[30] US National Institutes of Health Division of AIDS. *Division of AIDS Table for Grading the Severity of Adult and Pediatric Adverse Events*. 2009. http://rsc.tech-res.com/document/safetyandpharmacovigilance/table_for_grading_severity_of_adult_pediatric_adverse_events.pdf.

[31] Tietz N (1995). Clinical Guide to Laboratory Tests.

[32] Isanaka S, Mugusi F, Hawkins C, Spiegelman D, Okuma J, Aboud S, Guerino C, Fawzi WW (2012). Effect of high-dose vs standard-dose multivitamin supplementation at the initiation of HAART on HIV disease progression and mortality in Tanzania: a randomized controlled trial. JAMA..

[33] Schoenfeld D (1982). Partial residuals for the proportional hazards regression model. Biometrika..

[34] Filmer D, Pritchett L (2001). Estimating wealth effects without expenditure data – or tears: an application to educational enrolments in states of India. Demography..

[35] Koethe JR, Limbada MI, Giganti MJ, Nyirenda CK, Mulenga L, Wester CW, Chi BH, Stringer JS (2010). Early immunologic response and subsequent survival among malnourished adults receiving antiretroviral therapy in Urban Zambia. AIDS..

